# Supplementary material for: Developmental potential of aneuploid human embryos cultured beyond implantation
Source: Nat Commun. 2020 Aug 10;11:3987. doi: 10.1038/s41467-020-17764-7 (PMC7418029; doi:10.1038/s41467-020-17764-7)
Supplement: Supplementary file 1 — Supplementary Information [file 41467_2020_17764_MOESM1_ESM.pdf]

SUPPLEMENTARY INFORMATION

Developmental potential of aneuploid human embryos cultured beyond implantation

Shahbazi, Wang, Tao, Weatherbee, et al.

Supplementary Figure 1

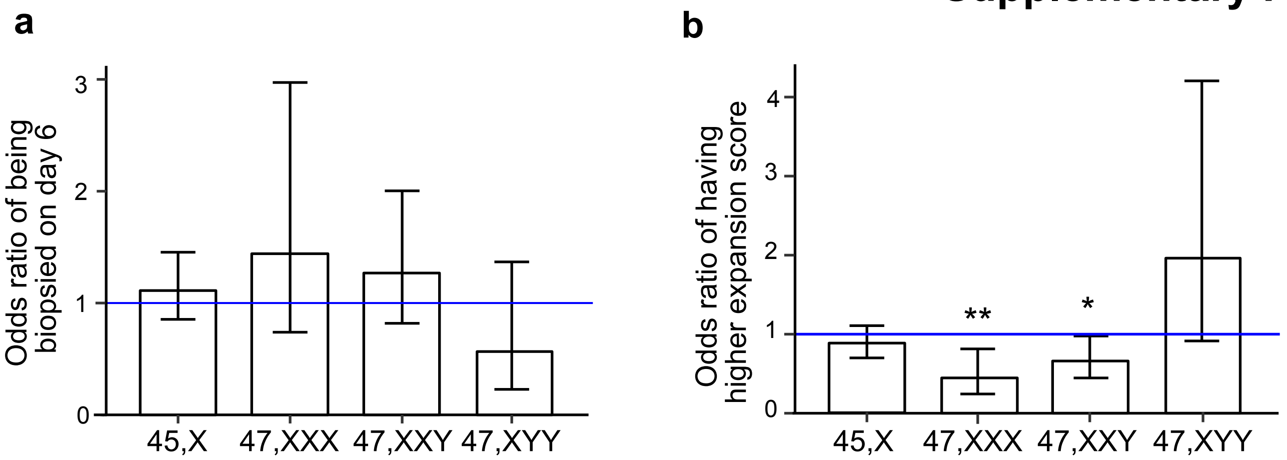

Supplementary Figure 1: Sex chromosome aneuploidies in pre-implantation human embryo. a, Odds ratios of embryos developing to the blastocyst stage by day 6 rather than day 5 for different sex chromosome aneuploidies as compared to euploid embryos. Error bars represent 95% profile likelihood confidence intervals. Blue line represents odds for euploid embryos. **b,** Odds ratios of embryos having a higher day 5 expansion score for different sex chromosome aneuploidies as compared to euploid embryos. Error bars are 95% profile likelihood confidence intervals. Blue line represents odds for euploid embryos. Confidence intervals, p-values and the specific number of embryos analyzed per genotype is shown in the Source Data file. A total of 374 sex chromosome aneuploid embryos were analysed. Likelihood ratio test (a) and Wald test of regression coefficient (b), *p<0.05, **p<0.01, ***p<0.001. Source data are provided as a Source Data file.

Supplementary Figure 2

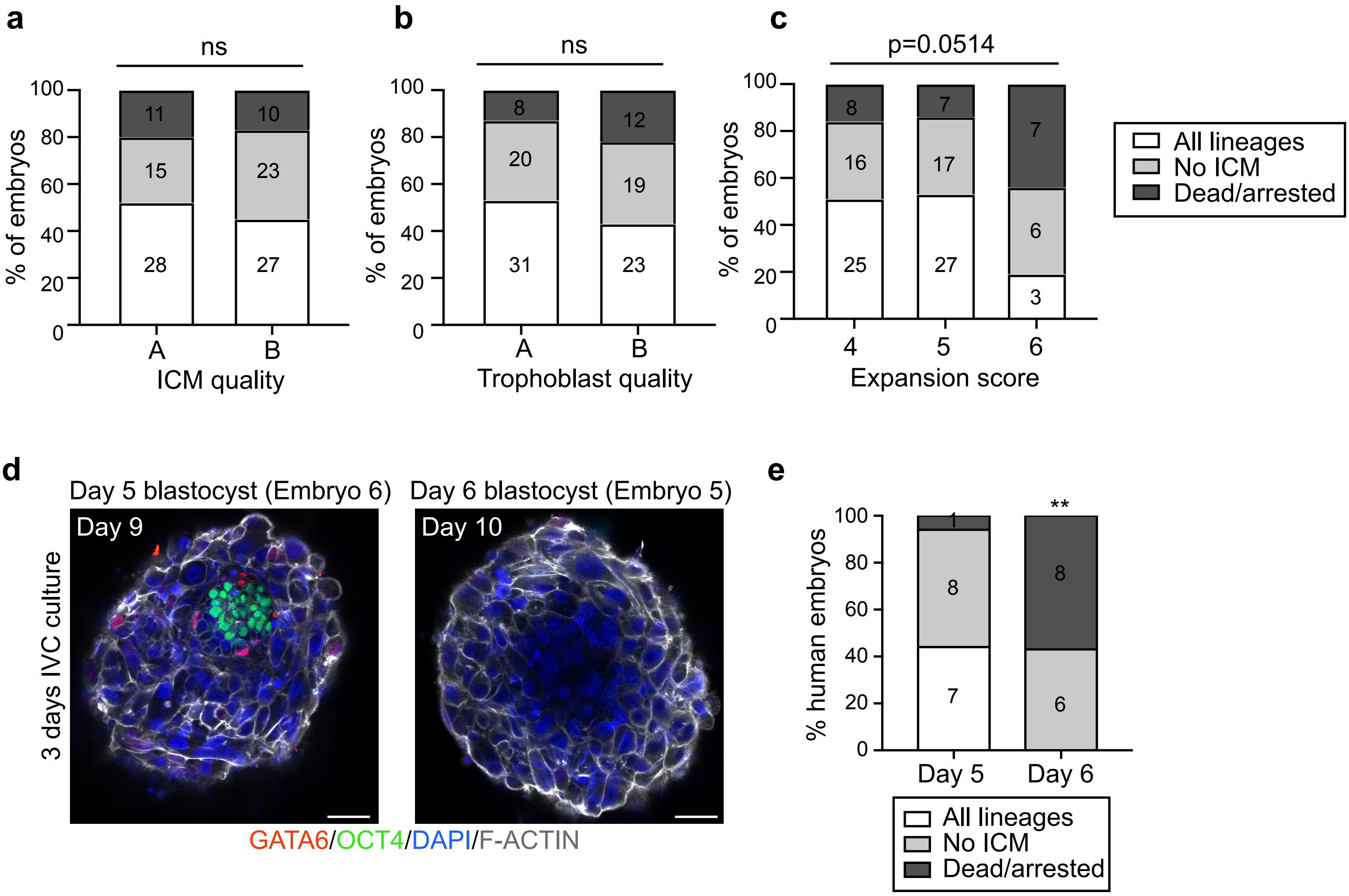

Supplementary Figure 2: Post-implantation *in vitro* development of blastocysts, classified according to their morphology and timing of development. a-c, Developmental phenotypes of embryos used in this study, classified according to the Gardner criteria. The number of embryos per category is indicated. Chi-square test, ns: non-significant. **d,** Immunostaining of human embryos cultured for 3 days in the IVC system. Representative images for each group are shown. Scale bars, 50 µm. **e,** Developmental phenotypes of embryos from panel d. The number of embryos per category is indicated. Chi-square test, **p=0.0018. 4 independent experiments. Source data are provided as a Source Data file.

Supplementary Figure 3

a

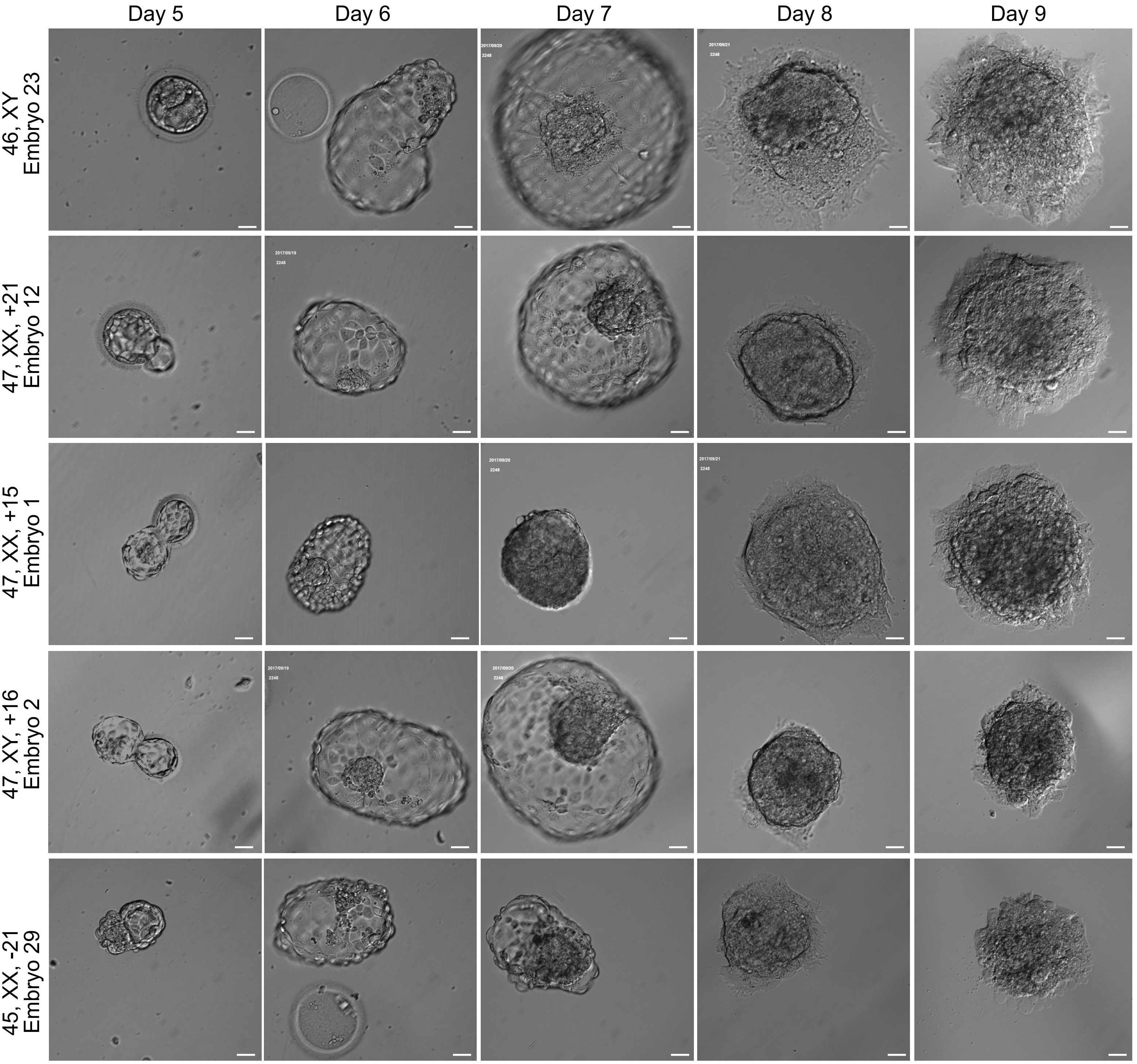

b

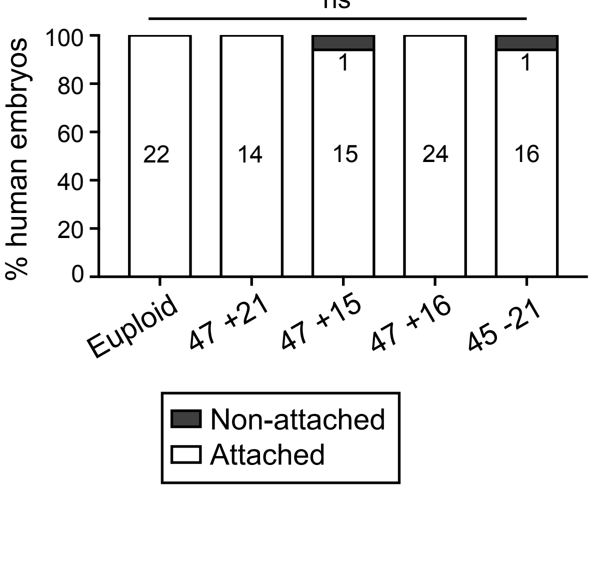

Supplementary Figure 3: Human embryo development *in vitro* at the pre- to post-implantation transition. a, Representative brightfield images of human embryos developing from day 5 to day 9 *in vitro*. **b,** Percentage of human embryos that attached or failed to attach during in vitro culture up to day 9. The number of embryos per category is indicated. Chi-square test, ns: non-significant. 4 independent experiments. Source data are provided as a Source Data file.

Supplementary Figure 4

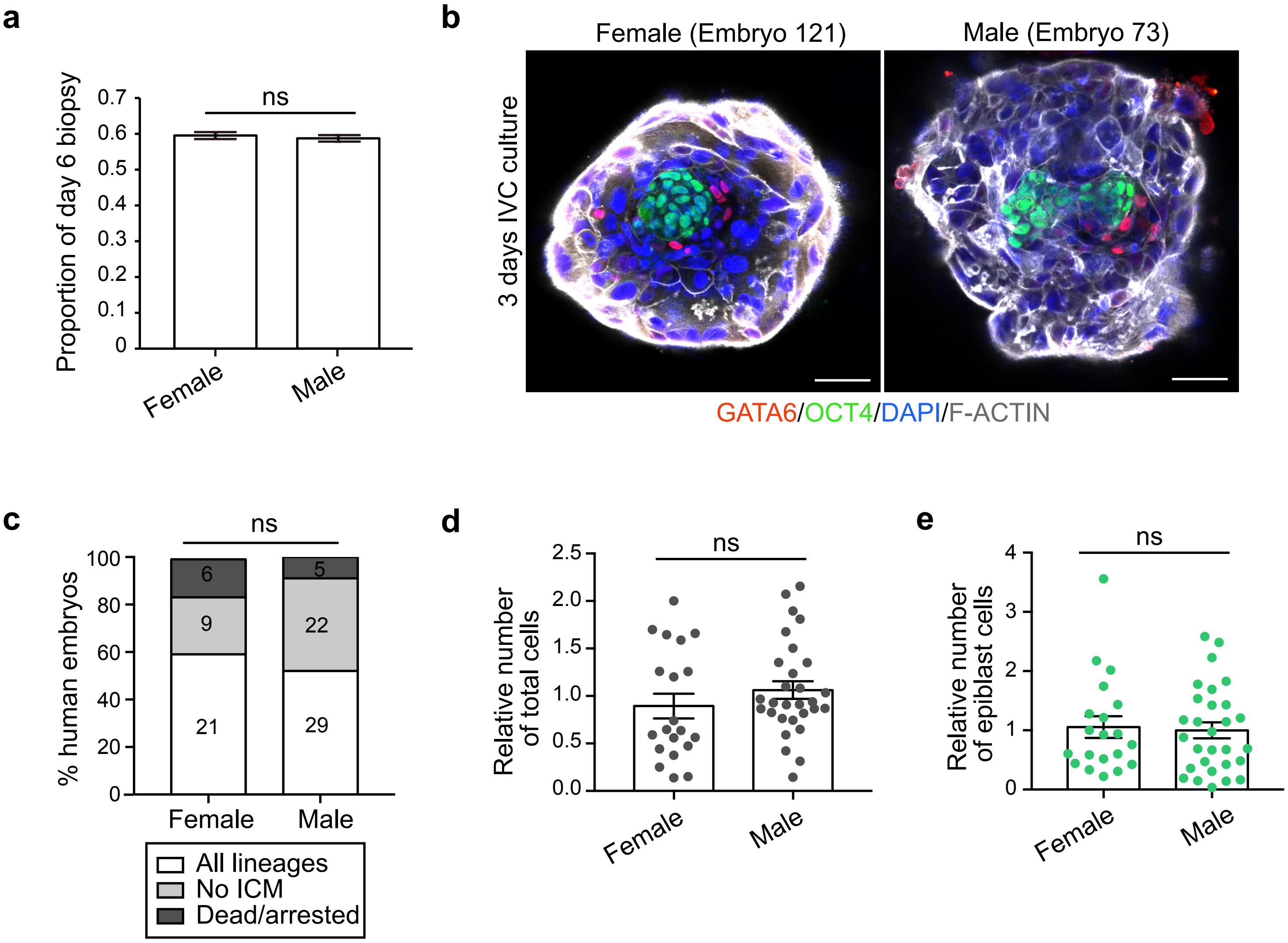

Supplementary Figure 4: Pre- and post-implantation *in vitro* development of female versus male embryos. a, Proportion of day 6 biopsy of euploid embryos (n = 25,368) in female and male. Logistic regression shows no significant difference (p=0.2). Bar, 95% binomial confidence interval. **b,** Immunostaining of human female and male embryos cultured for 3 days in the IVC system. Representative images for each group are shown. Scale bars, 50 µm. **c,** Developmental phenotypes of embryos from panel b. The number of embryos per category is indicated. Chi-square test, ns: non-significant. **d,** Total number of cells per embryo, relative to the corresponding karyotype, in embryos from panel b. Each dot represents an individual embryo. n = 20 female and 29 male embryos. Unpaired Student's t-test, ns: non-significant. **e,** Number of epiblast cells per embryo, relative to the corresponding karyotype, in embryos from panel b. Each green dot represents an individual embryo. n = 20 female and 29 male embryos. Unpaired Student's t-test, ns: non-significant. All error bars represent s.e.m. 4 independent experiments. Source data are provided as a Source Data file.

Supplementary Figure 5

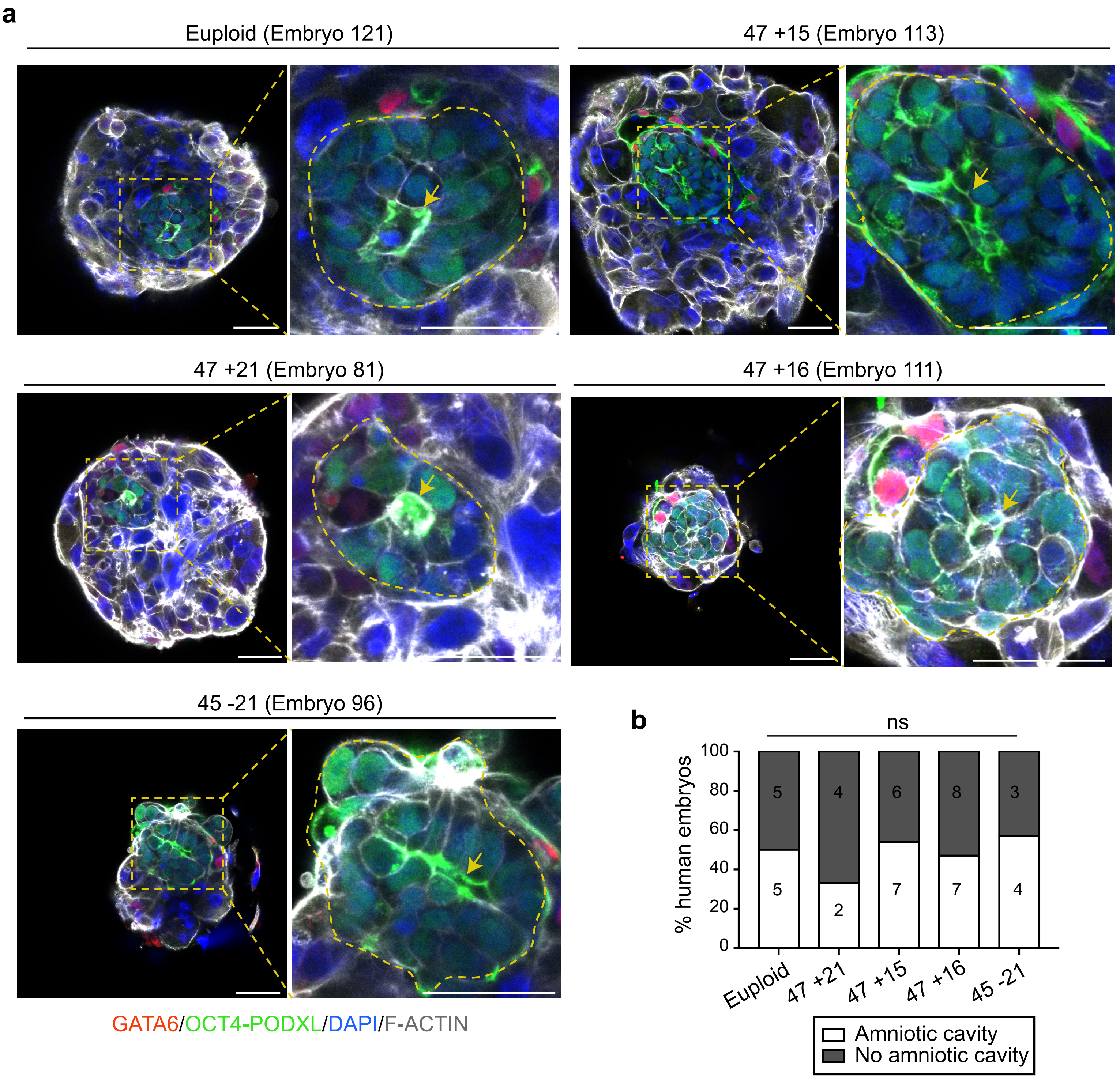

Supplementary Figure 5: Amniotic cavity formation in embryos cultured *in vitro* up to day 9. a, Immunostaining of human embryos cultured for 3 days in the IVC system. Representative images for each aneuploidy are shown. Squares denote magnified regions. Dotted lines mark the epiblast. Arrows mark the amniotic cavity. Scale bars, 50 µm. **b,** Amniotic cavity formation in embryos from panel a. The number of embryos per category is indicated. Chi-square test, ns: non-significant. 4 independent experiments. Source data are provided as a Source Data file.

Supplementary Figure 6

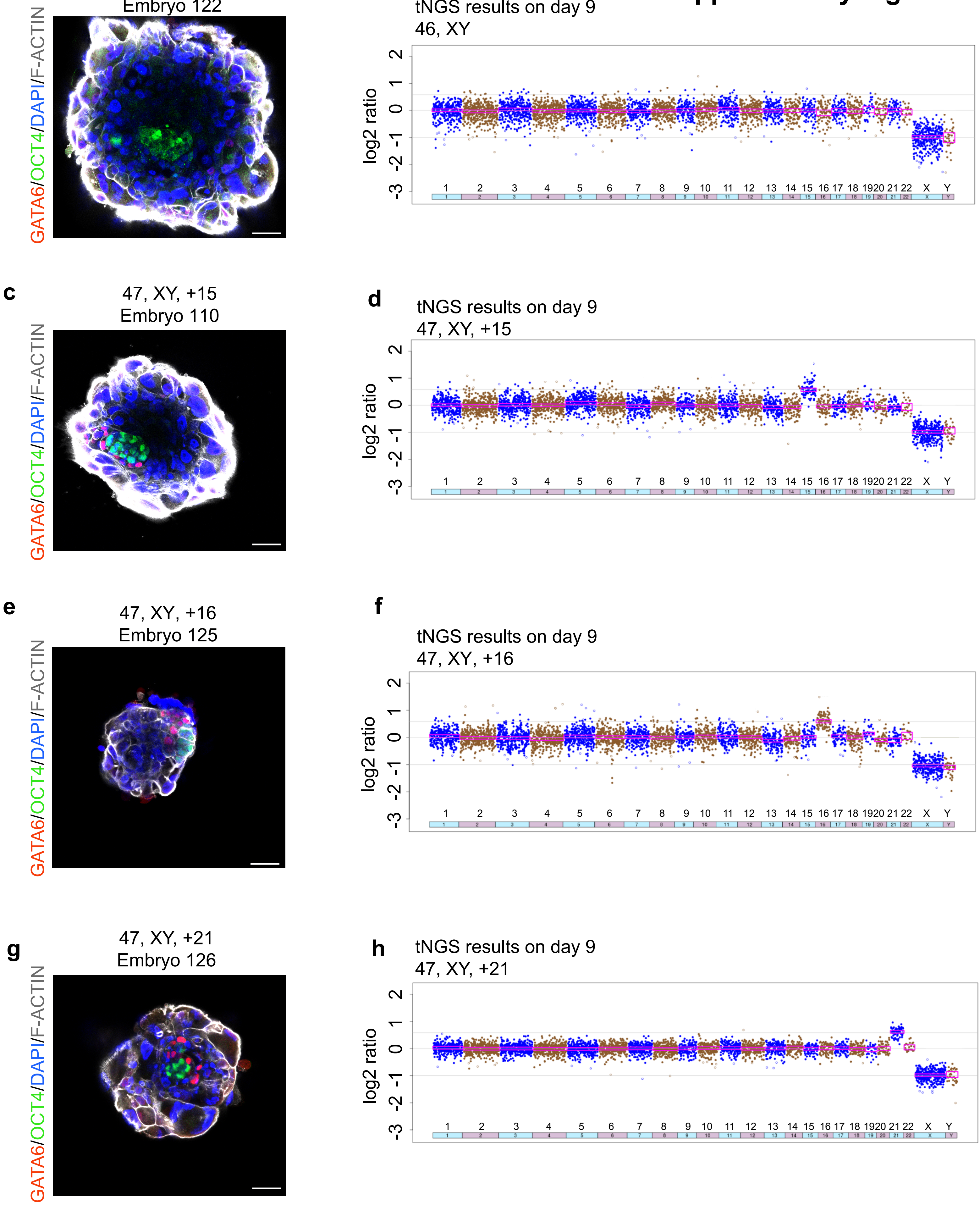

Supplementary Figure 6: Representative chromosome copy number analysis of post-implantation embryos cultured in vitro until day 9. a, Immunostaining of a day 9 euploid embryo (#122). **b,** Chromosome copy number analysis from the dissections of the fixed euploid embryo (46,XY). **c,** Immunostaining of a day 9 trisomy 15 embryo (#110). **d,** Chromosome copy number analysis from the dissections of the fixed embryo (47,XY,+15). **e,** Immunostaining of a day 9 trisomy 16 embryo (#125). **f,** Chromosome copy number analysis from the dissections of the fixed embryo (47,XY,+16). **g,** Immunostaining of a day 9 trisomy 21 embryo (#126). **h,** Chromosome copy number analysis from the dissections of the fixed embryo (47,XY,+21). Scale bars, 50 µm.

Supplementary Figure 7

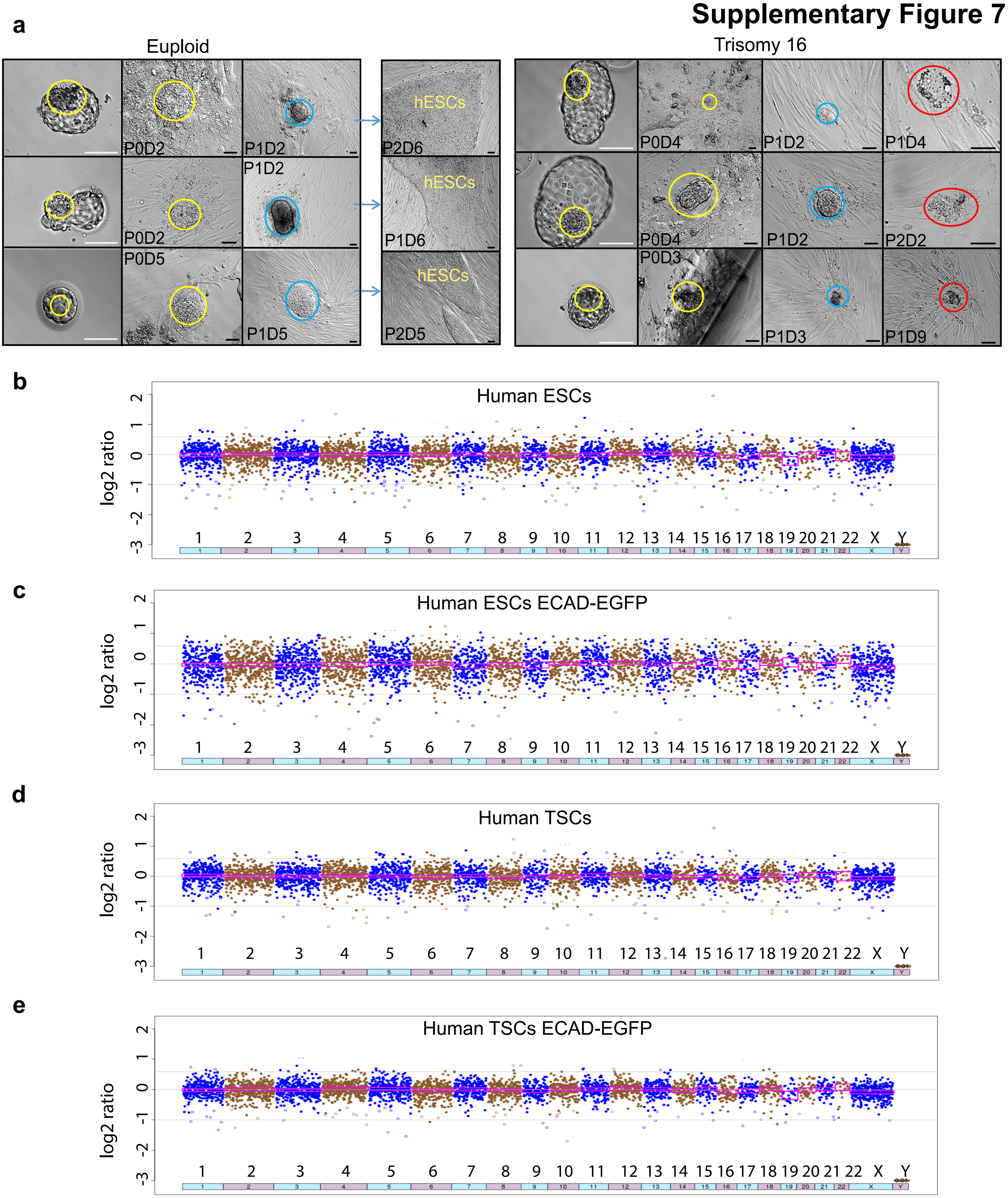

Supplementary Figure 7: Chromosome copy number analysis of stem cell lines. a, Composite micrographs of human embryos and attempted derivation of human ESCs over time. Three warmed day 5 human blastocysts with PGT-A diagnosis of trisomy 16 and three with PGT-A indication of euploidy were used to attempt human ESC derivation 23 hours post-warming. Early ICMs attached (yellow circles) during passage 0 (P0) days 2-5 (D2-5) and developed to early epiblast-like structures (blue circles) by P1D2-5. During growth in P1, trisomy 16-epiblast-like structures ceased growing and degenerated (red circles), whereas euploid-epiblast-like structures continued expansion with development of human ESC colonies and established human ESC lines. White scale bars, 100 µm. Black scale bars, 10 µm. **b,** NGS results of H9 human ESCs (46, XX). **c,** NGS results of H9 human ESCs transfected with a CDH1-EGFP expressing plasmid (46, XX). **d,** NGS results of human TSCs (46, XX). **e,** NGS results of human TSCs transfected with a CDH1-EGFP expressing plasmid (46, XX).

Supplementary Figure 8

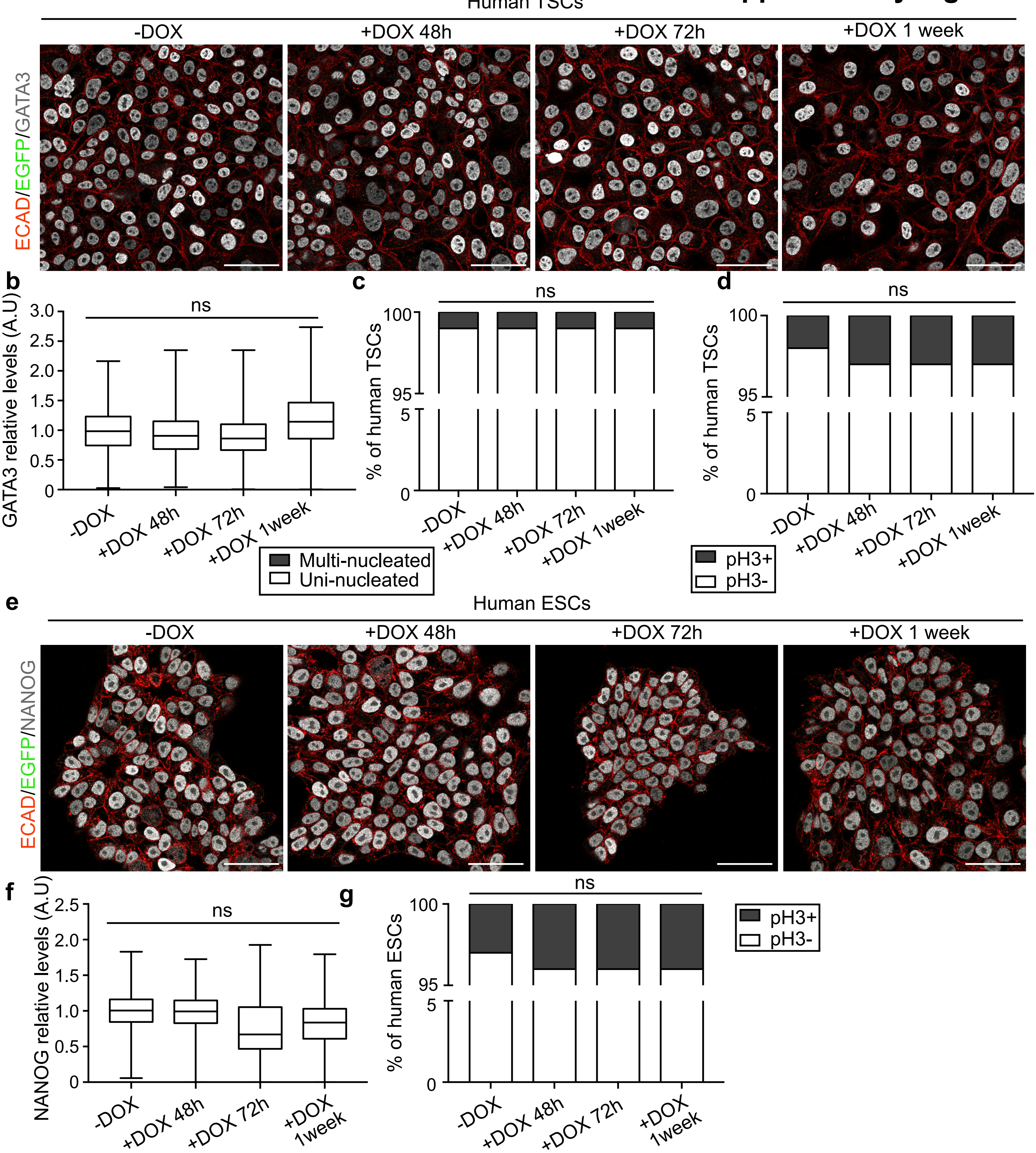

Supplementary Figure 8: DOX administration does not affect human TSC and ESC proliferation and stemness. **a,** Immunostaining of human TSCs in the presence or absence of DOX. **b,** Quantification of GATA3 levels in cells from panel a. n = 2,689, 2,516, 1,924 and 1,849 cells per condition. Kruskal Wallis test, ns: non-significant. Data is shown in a box plot. Whiskers go from minimum to maximum values. The box extends from the 25th to 75th percentile, and the middle line represents the median. **c,** Percentage of uni-nucleated and multi-nucleated cells in cells from panel a. n = 2,082, 539, 241 and 109 cells per condition. Chi-square test, ns: non-significant. **d,** Percentage of phospho-HISTONE H3 (pH3) positive cells in cells from panel a. n = 2,631, 2,462, 1,889 and 1,838 cells per condition. Chi-square test, ns: non-significant. **e,** Immunostaining of human ESCs in the presence or absence of DOX. **f,** Quantification of NANOG levels in cells from panel e. n = 1,557, 1,199, 2,109 and 1,260 cells per condition. Kruskal Wallis test, ns: non-significant. Data is shown in a box plot. Whiskers go from minimum to maximum values. The box extends from the 25th to 75th percentile, and the middle line represents the median. **g,** Percentage of phospho-HISTONE H3 (pH3) positive cells in cells from panel e. Chi-square test, ns: non-significant. n = 1,274, 2,054, 1,059 and 1,411 cells per condition. All scale bars, 50 μm. 3 independent experiments. Source data are provided as a Source Data file.

Supplementary Figure 9

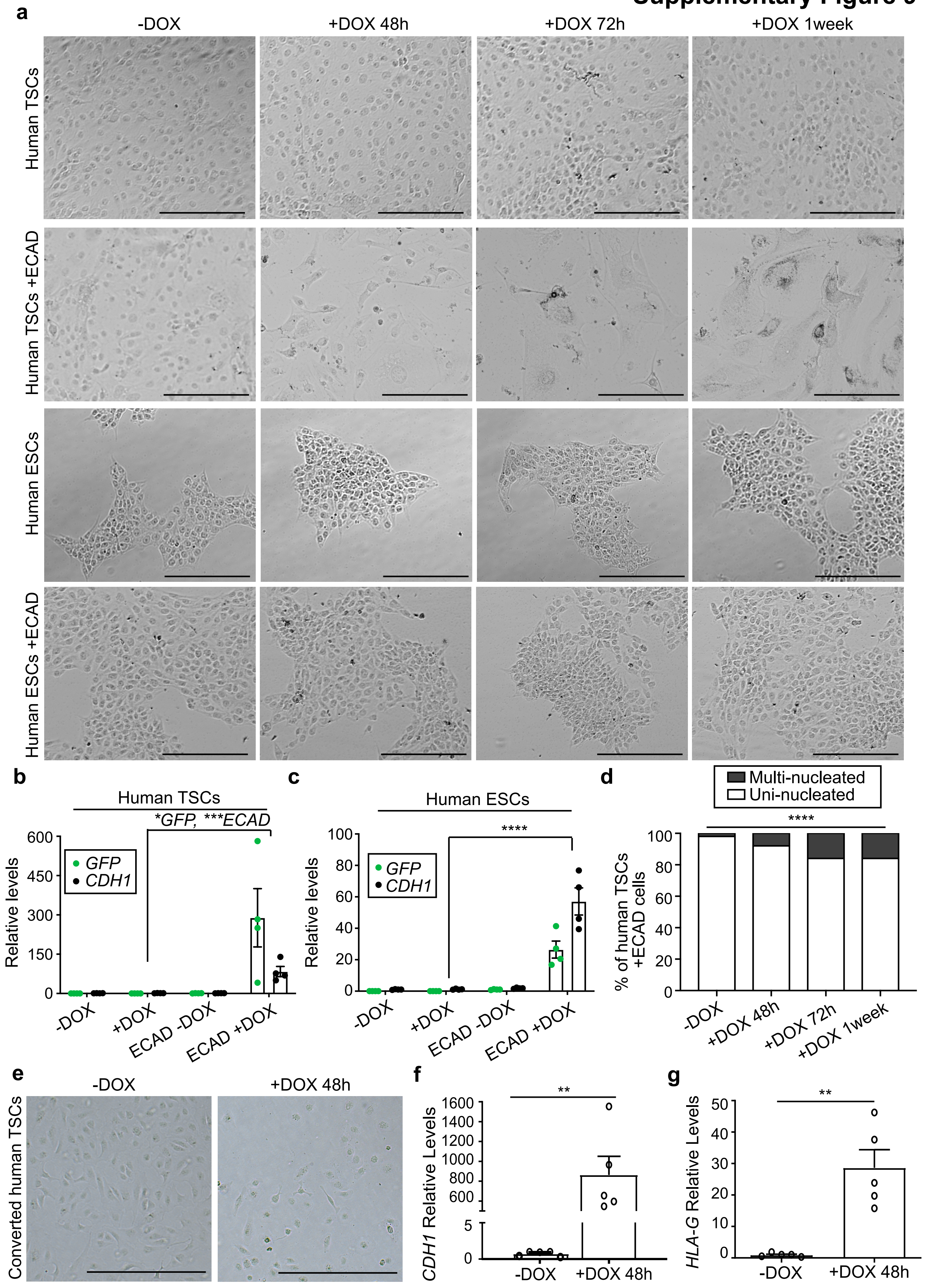

Supplementary Figure 9: Characterization of ECAD-overexpressing human TSCs and ESCs. a, Representative brightfield images of human ESCs and human TSCs transfected/non-transfected with a plasmid expressing CDH1-EGFP in the presence or absence of DOX. **b-c,** RT-PCR analysis of GFP and CDH1 levels in human ESCs and TSCs that were/were not transfected with a CDH1-EGFP expressing plasmid in the presence or absence of DOX. Each dot represents one sample. n = 4 samples per condition. One-way ANOVA with a multiple comparisons test, *p<0.0147, ***p=0.0003, ****p<0.0001, ns. **d,** Percentage of uni-nucleated and multi-nucleated cells in TSCs from Fig. 4a. n = 2,082, 539, 241 and 109 cells per condition. Chi-square test, ****p<0.0001. **e,** Brightfield images of converted TSCs with and without DOX administration. **f-g,** RT-PCR of CDH1 and HLA-G in converted TSCs with and without administration of DOX. Each dot represents one sample. n = 5 samples per condition. Unpaired Student's t-test, **p=0.001 (CDH1) and **p=0.0084 (HLA-G). All error bars represent s.e.m. 3 independent experiments (panel a-d) and 2 independent experiments (e-g). All scale bars, 500 µm. Source data are provided as a Source Data file.

	EXPERIMENT 1	EXPERIMENT 2	EXPERIMENT 3	EXPERIMENT 4	EXPERIMENT 5	TOTAL
Embryos thawed	33	40	36	34	20	163
Embryos lost during culture	1	0	1	0	0	2
Embryos dead/inside zona before culture	5	7	12	2	0	26
Embryos analyzed	27	33	23	32	20	135

Supplementary Table 1: Summary of all the experiments that involved culture of human embryos *in vitro* up to day 9.

Exp No.	CCS ID	Embryo ID	Test blastocyst	Karyotype	Biopsy day	Biopsy day			Outcome	Test day9	Karyotype	Match
						Expansion	ICM	TE				
1	59979a1	1	NexCCS	47,XX,+15	5	4	A	A	Differentiated			
	59772a8	2	NexCCS	47,XY,+16	5	5	B	A	All lineages			
	59803a1	3	NexCCS	47,XX,+18	6	4	A	A	Dead/inside zona before culture			
	59965a21	4	NexCCS	47,XY,+22	5	4	A	A	Differentiated			
	59919a11	5	NexCCS	47,XY,+15	6	6	B	B	Differentiated			
	59975a3	6	NexCCS	47,XX,+15	5	5	A	A	All lineages			
	59725a9	7	NexCCS	47,XY,+15	5	4	B	A	All lineages			
	59962a1	8	NexCCS	47,XY,+16	5	4	A	A	Differentiated			
	59757a1	9	NexCCS	47,XY,+22	5	4	A	A	All lineages			
	59649a9	10	NexCCS	47,XY,+22	6	6	B	B	Dead/arrested during culture			
	59844a10	11	NexCCS	47,XY,+22	6	5	A	A	Differentiated			
	59918a7	12	NexCCS	47,XX,+21	5	4	B	B	Differentiated			
	59106a1	13	NexCCS	47,XY,+16	6	6	B	B	Dead/arrested during culture			
	59106a14	14	NexCCS	47,XX,+16	5	4	A	A	All lineages			
	52419a6	15	NexCCS	47,XY,+18	6	5	B	B	Dead/arrested during culture			
	57844a11	16	NexCCS	47,XX,+21	6	4	B	B	Dead/inside zona before culture			
	58971a1	17	NexCCS	47,XY,+21	5	5	B	A	Differentiated			
	52764a4	18	NexCCS	47,XX,+18	5	5	B	B	Differentiated			
	58199a6	19	NexCCS	47,XX,+21	6	6	B	A	Differentiated			
	52639a7	20	NexCCS	47,XX,+18	6	6	B	B	Dead/arrested during culture			
	57270a2	21	PCR-24	46,XY	5	4	B	C	Dead/inside zona before culture			
	33796a1	22	PCR-24	46,XX	6	6	B	A	Dead/arrested during culture			
	57270a1	23	PCR-24	46,XY	5	5	B	A	Differentiated			

	57862a1	24	NexCCS	47,XX,+18	6	6	B	B	Dead/arrested during culture			
	33796a7	25	PCR-24	46,XX	6	6	C	C	Lost			
	59842a5	26	NexCCS	45,XY,-11	5	5	B	B	All lineages			
	59823a1	27	NexCCS	45,XY,-21	5	4	A	B	Differentiated			
	59772a26	28	NexCCS	45,XY,-22	6	5	B	B	Differentiated			
	59752a1	29	NexCCS	45,XX,-21	6	6	B	B	Differentiated			
	59864a1	30	NexCCS	45,XX,-21	5	4	A	B	Dead/inside zona before culture			
	59732a19	31	NexCCS	45,XX,-7	5	5	A	A	All lineages			
	59675a6	32	NexCCS	45,XY,-18	6	4	B	B	Dead/inside zona before culture			
	59675a18	33	NexCCS	45,XY,-22	5	4	A	A	Dead/arrested during culture			
2	59432a3	34	NexCCS	47,XX,+16	5	4	A	B	All lineages			
	52764a10	35	NexCCS	45,XY,-21	5	4	B	B	Differentiated			
	56797a1	36	NexCCS	47,XY,+15	5	4	A	A	All lineages			
	58258a3	37	NexCCS	47,XY,+21	5	6	B	B	Differentiated			
	57811a2	38	NexCCS	47,XX,+16	5	4	A	B	Dead/arrested during culture			
	56022a4	39	NexCCS	47,XX,+16	5	5	A	A	Dead/inside zona before culture			
	56075a1	40	NexCCS	45,XY,-21	5	5	B	B	Dead/inside zona before culture			
	57493a1	41	NexCCS	47,XY,+16	5	5	B	A	Differentiated			
	57610a3	42	NexCCS	47,XX,+15	5	4	B	B	All lineages			
	57520a10	43	NexCCS	45,XY,-21	5	6	B	B	All lineages			
	55600a14	44	NexCCS	47,XY,+15	5	5	A	A	All lineages			
	53503a2	45	NexCCS	47,XX,+15	5	5	B	A	All lineages			
	56423a4	46	NexCCS	47,XX,+21	5	5	A	B	All lineages			
	54470a5	47	NexCCS	45,XY,-21	5	5	B	B	All lineages			
	60860a5	48	NexCCS	45,XX,-21	5	5	A	A	Dead/arrested during culture			

	55890a17	49	NexCCS	45,XX,-11	5	4	A	B	Dead/inside zona before culture			
	55800a2	50	NexCCS	45,XY,-21	5	4	A	A	All lineages			
	52396a7	51	NexCCS	47,XY,+15	5	5	A	A	Differentiated			
	55101a14	52	NexCCS	45,XX,-11	5	5	A	A	Dead/arrested during culture			
	56460a2	53	NexCCS	45,XY,-11	5	4	A	B	Dead/arrested during culture			
	59432a1	54	NexCCS	47,XY,+16	5	4	A	A	All lineages			
	57495a1	55	NexCCS	45,XX,-21	5	5	A	A	Differentiated			
	59447a2	56	NexCCS	47,XX,+21	5	4	A	B	Dead/inside zona before culture			
	54700a1	57	NexCCS	45,XX,-11	5	5	A	A	Dead/inside zona before culture			
	60545a5	58	NexCCS	47,XX,+15	5	4	A	A	Differentiated			
	52311a5	59	NexCCS	47,XY,+21	5	5	A	A	All lineages			
	55174a2	60	NexCCS	47,XX,+16	5	5	A	A	Differentiated			
	52783a1	61	NexCCS	47,XX,+16	5	4	B	B	Differentiated			
	58591a5	62	NexCCS	47,XY,+21	5	5	A	A	All lineages			
	54700a2	63	NexCCS	47,XY,+21	5	4	B	A	Differentiated			
	55082a1	64	NexCCS	47,XY,+21	5	5	B	A	Dead/inside zona before culture			
	59066a1	65	NexCCS	47,XX,+15	5	5	B	B	Dead/inside zona before culture			
	50659a7	66	PCR-24	46,XY	5	5	B	A	Differentiated			
	50277a19	67	PCR-24	46,XY	5	5	B	B	Differentiated			
	50277a18	68	PCR-24	46,XY	5	5	A	B	Differentiated			
	50277a17	69	PCR-24	46,XX	5	5	B	B	Differentiated			
	50277a13	70	PCR-24	46,XY	5	5	B	B	Differentiated			
	52396a2	71	NexCCS	46,XX	5	4	A	A	All lineages			
	52396a8	72	NexCCS	46,XY	5	4	A	A	Differentiated			
	52396a10	73	NexCCS	46,XY	5	5	A	A	All lineages			
3	63843a5	74	NexCCS	47,XX,+15	5	5	B	A	All lineages	NexCCS	47,XX,+15	Yes

	61854a10	75	NexCCS	47,XY,+16	5	4	A	B	Dead/inside zona before culture			
	59134a18	76	NexCCS	45,XX,-21	5	4	A	A	Dead/inside zona before culture			
	57831a10	77	NexCCS	45,XY,-21	5	5	A	B	Dead/arrested during culture			
	62821a1	78	NexCCS	47,XX,+15	5	4	B	B	All lineages			
	62379a77	79	NexCCS	47,XY,+16	5	5	B	B	All lineages	NexCCS	NA	
	60974a1	80	NexCCS	47,XY,+16	5	5	B	B	All lineages			
	53588a9	81	NexCCS	47,XX,+21	5	5	B	B	All lineages			
	56797a16	82	NexCCS	47,XX,+21	5	4	A	A	Dead/arrested during culture			
	62582a15	83	NexCCS	47,XX,+16	5	5	B	B	Dead/inside zona before culture	NexCCS	47,XX,+16	Yes
	63650a7	84	NexCCS	45,XX,-21	5	5	B	A	All lineages			
	61250a1	85	NexCCS	45,XX,-21	5	4	B	B	Dead/arrested during culture			
	52464a7	86	NexCCS	47,XX,+21	5	4	A	A	Dead/arrested during culture	NexCCS	Mosaic (47,XX,+21; 45,XX,-21)	No
	61657a11	87	NexCCS	47,XY,+16	5	5	B	A	Dead/inside zona before culture			
	62750a13	88	NexCCS	45,XY,-21	5	5	A	A	Dead/arrested during culture			
	63807a10	89	NexCCS	45,XX,-21	5	4	B	B	All lineages	NexCCS	Mosaic (45,XX,-21; 46,XX)	No
	62211a16	90	NexCCS	47,XX,+21	5	4	A	A	All lineages			
	62575a1	91	NexCCS	47,XY,+16	5	4	A	B	All lineages			
	64034a8	92	NexCCS	45,XX,-21	5	5	B	B	Lost			
	56423a7	93	NexCCS	47,XY,+21	5	5	B	B	Dead/inside zona before culture			
	62575a5	94	NexCCS	45,XY,-21	5	4	B	B	Dead/arrested during culture			

	58611a3	95	NexCCS	47,XY,+15	5	5	A	A	All lineages	NexCCS	47,XY,+15	Yes
	60222a5	96	NexCCS	45,XY,-21	5	4	A	A	All lineages	NexCCS	NA	
	55760a10	97	NexCCS	45,XX,-21	5	4	B	B	Dead/inside zona before culture			
	59522a23	98	NexCCS	47,XX,+16	5	4	B	B	Dead/inside zona before culture			
	63109a17	99	NexCCS	47,XY,+21	5	5	B	B	Differentiated	NexCCS	47,XY,+21	Yes
	60606a17	100	NexCCS	47,XX,+16	5	5	B	A	All lineages	NexCCS	NA	
	62514a7	101	NexCCS	47,XX,+16	5	4	B	A	All lineages	NexCCS	46,XX	No
	41277a3	102	PCR-24	46,XY	5	5	A	A	Dead/inside zona before culture			
	41785a5	103	PCR-24	46,XY	5	6	B	B	Differentiated			
	47572a5	104	PCR-24	46,XX	5	6	B	A	All lineages	NexCCS	46,XX	Yes
	50857a4	105	PCR-24	46,XY	5	5	C	B	Dead/inside zona before culture			
	48071a4	106	PCR-24	46,XY	5	4	B	C	Dead/arrested during culture			
	46815a10	107	PCR-24	46,XX	5	6	B	B	Dead/inside zona before culture			
	47428a29	108	PCR-24	46,XX	5	6	B	C	Dead/inside zona before culture			
	47428a22	109	PCR-24	46,XX	5	6	C	B	Dead/inside zona before culture			
4	65333a2	110	NexCCS	47,XY,+15	5	4	A	A	All lineages	NexCCS	47,XY,+15	Yes
	64446a38	111	NexCCS	47,XY,+16	5	4	A	B	All lineages	NexCCS	47,XY,+16	Yes
	64324a13	112	NexCCS	47,XX,+16	5	4	A	B	All lineages	NexCCS	47,XX,+16	Yes
	64446a53	113	NexCCS	47,XY,+15	5	4	A	A	All lineages			
	55812a2	114	NexCCS	46,XX	5	5	B	A	Dead/inside zona before culture			
	55812a1	115	NexCCS	46,XY	5	5	A	A	All lineages	NexCCS	46,XY	Yes
	55812a3	116	NexCCS	46,XY	5	4	B	A	Differentiated	NexCCS	46,XY	Yes
	58703a3	117	NexCCS	46,XY	5	4	B	A	All lineages	NexCCS	46,XY	Yes
	59707a8	118	NexCCS	46,XY	5	5	A	A	Differentiated	NexCCS	46,XY	Yes
	58021a5	119	NexCCS	46,XY	5	5	B	B	Differentiated	NexCCS	46,XY	Yes

	58021a5	120	NexCCS	46,XY	5	5	B	B	All lineages	NexCCS	46,XY	Yes
	54470a9	121	NexCCS	46,XX	5	4	B	C	All lineages			
	54470a1	122	NexCCS	46,XY	5	5	B	B	All lineages	NexCCS	46,XY	Yes
	54470a4	123	NexCCS	46,XY	5	5	B	B	All lineages			
	52663a7	124	NexCCS	46,XY	5	5	A	A	All lineages	NexCCS	46,XY	Yes
	63982a1	125	NexCCS	47,XY,+16	5	5	B	B	All lineages	NexCCS	47,XY,+16	Yes
	62279a18	126	NexCCS	47,XY,+21	5	4	B	B	All lineages	NexCCS	47,XY,+21	Yes
	62087a2	127	NexCCS	47,XY,+15	5	5	B	B	All lineages	NexCCS	47,XY,+15	Yes
	58413a8	128	NexCCS	47,XX,+16	5	4	A	A	Differentiated	NexCCS	47,XX,+16	Yes
	58658a31	129	NexCCS	47,XX,+16	5	4	A	A	Differentiated	NexCCS	47,XX,+16	Yes
	63848a15	130	NexCCS	47,XY,+16	5	5	B	B	All lineages	NexCCS	47,XY,+16	Yes
	62526a9	131	NexCCS	47,XX,+15	5	4	A	A	All lineages			
	58658a16	132	NexCCS	47,XX,+16	5	5	A	B	All lineages			
	49869a7	133	Microarray	47,XY,+21	5	4	B	A	Differentiated	NexCCS	47,XY,+21	Yes
	65682a9	134	NexCCS	47,XX,+16	5	4	A	A	All lineages			
	65943a9	135	NexCCS	47,XY,+16	5	4	B	B	Differentiated	NexCCS	47,XY,+16	Yes
	65822a5	136	NexCCS	47,XY,+16	5	4	A	B	Differentiated	NexCCS	47,XY,+16	Yes
	XIN-1	137	PCR-24	47,XY,+21	6	6	B	C	Dead/arrested during culture			
	XIN-2	138	PCR-24	47,XX,+21	6	6	C	B	Dead/arrested during culture			
	XIN-3	139	PCR-24	47,XY,+21	6	6	C	B	Differentiated			
	65891a2	140	NexCCS	45,XY,-21	5	4	B	B	Dead/inside zona before culture			
	53884a10	141	Microarray	45,XX,-21	5	5	A	A	Dead/arrested during culture	NexCCS	NA	
	65549a29	142	NexCCS	45,XY,-21	5	5	A	B	Dead/arrested during culture	NexCCS	45,XY,-21	Yes
	65612a1	143	NexCCS	45,XX,-21	5	4	A	B	All lineages	NexCCS	NA	
5	62253a16	144	NexCCS	46,XX	5	5	A	A	ECAD/SDC1/pH3 analysis			
	48644a2	145	NexCCS	46,XY	5	4	B	B	ECAD/SDC1/pH3 analysis			

	48644a5	146	NexCCS	46,XY	5	5	B	B	ECAD/SDC1/pH3 analysis			
	56577a1	147	NexCCS	46,XX	5	5	B	A	ECAD/SDC1/pH3 analysis			
	48644a18	148	NexCCS	46,XX	5	4	B	A	ECAD/SDC1/pH3 analysis			
	45799a3	149	NexCCS	46,XY	5	5	B	B	ECAD/SDC1/pH3 analysis			
	45799a1	150	NexCCS	46,XX	5	5	B	A	ECAD/SDC1/pH3 analysis			
	44527a1	151	NexCCS	46,XY	5	5	A	B	ECAD/SDC1/pH3 analysis			
	66522a8	152	NexCCS	47,XY,+16	5	5	B	B	ECAD/SDC1/pH3 analysis			
	68188a5	153	NexCCS	47,XY,+16	5	5	A	A	ECAD/SDC1/pH3 analysis			
	67545a2	154	NexCCS	47,XX,+16	5	4	B	B	ECAD/SDC1/pH3 analysis			
	67798a26	155	NexCCS	47,XY,+16	5	5	B	B	ECAD/SDC1/pH3 analysis			
	67805a21	156	NexCCS	47,XY,+16	6	6	B	A	Dead/arrested during culture			
	68691a27	157	NexCCS	47,XX,+16	5	4	B	B	ECAD/SDC1/pH3 analysis			
	68621a18	158	NexCCS	47,XY,+16	5	5	B	A	ECAD/SDC1/pH3 analysis			
	65423a29	159	NexCCS	47,XY,+16	5	5	C	B	ECAD/SDC1/pH3 analysis			
	42433a2	160	NexCCS	46,XX	5	5	B	B	Dead/arrested during culture			
	49296a9	161	NexCCS	46,XY	5	5	B	B	Dead/arrested during culture			
	67405a1	162	NexCCS	47,XY,+16	6	4	B	B	Dead/arrested during culture			
	68025a1	163	NexCCS	47,XX,+16	6	5	B	B	Dead/arrested during culture			

Supplementary Table 2: Blastocysts thawed in this study including their morphological and genetic assessment at day 5/6 and 9. Embryos highlighted in blue were taken into consideration for the analyses shown in Fig. 2d to g and Supplementary Fig. 5.

GENE	FW PRIMER	RV PRIMER	SOURCE
AXIN2	GCGATCCTGTTAATCCTTATCAC	AATTCCATCTACACTGCTGTC	Hwang et al 2014
CDH1	ATTTTTCCCTCGACACCCGAT	TCCCAGGCGTAGACCAAGA	Harvard Primer Bank
GFP	AAGGGCATCGACTTCAAGG	TGCTTGTCGGCCATGATATAG	Jackson Laboratories
HLA-G	GAGGAGACACGGAACACCAAG	TCGCAGCCAATCATCCACT	Shao et al
HPRT	TGACACTGGCAAAACAATGCA	GGTCCTTTTCACCAGCAAGCT	Gafni et al
NANOG	GATTTGTGGGCCTGAAGAAA	CAGATCCATGGAGGAAGGAA	Gafni et al
POU5F1	AGTGATTCTCCTGCCTCAGC	CTTCTGCTTCAGGAGCTTGG	Gafni et al
SDC1	CTATTCCCACGTCTCCAGAACC	GGACTACAGCCTCTCCCTCCTT	Okae et al

Supplementary Table 3: RT-PCR primers used in this study.
