# Supplementary material for: Developmental potential of aneuploid human embryos cultured beyond implantation
Source: Nat Commun. 2020 Aug 10;11:3987. doi: 10.1038/s41467-020-17764-7 (PMC7418029; doi:10.1038/s41467-020-17764-7)
Supplement: Supplementary file 3 — Reporting Summary [file 41467_2020_17764_MOESM3_ESM.pdf]

nature research

Corresponding author(s): Richard T. Scott Jr., Emre Seli and Magdalena Zernicka-Goetz

Last updated by author(s): 17/06/20

Reporting Summary

Nature Research wishes to improve the reproducibility of the work that we publish. This form provides structure for consistency and transparency in reporting. For further information on Nature Research policies, seeAuthors & Referees and theEditorial Policy Checklist .

Statistics

For all statistical analyses, confirm that the following items are present in the figure legend, table legend, main text, or Methods section.

n/a	Confirmed	
☐	☒	The exact sample size (*n*) for each experimental group/condition, given as a discrete number and unit of measurement
☐	☒	A statement on whether measurements were taken from distinct samples or whether the same sample was measured repeatedly
☐	☒	The statistical test(s) used AND whether they are one- or two-sided<br>*Only common tests should be described solely by name; describe more complex techniques in the Methods section.*
☒	☐	A description of all covariates tested
☐	☒	A description of any assumptions or corrections, such as tests of normality and adjustment for multiple comparisons
☐	☒	A full description of the statistical parameters including central tendency (e.g. means) or other basic estimates (e.g. regression coefficient) AND variation (e.g. standard deviation) or associated estimates of uncertainty (e.g. confidence intervals)
☐	☒	For null hypothesis testing, the test statistic (e.g. *F*, *t*, *r*) with confidence intervals, effect sizes, degrees of freedom and *P* value noted<br>*Give P values as exact values whenever suitable.*
☒	☐	For Bayesian analysis, information on the choice of priors and Markov chain Monte Carlo settings
☒	☐	For hierarchical and complex designs, identification of the appropriate level for tests and full reporting of outcomes
☒	☐	Estimates of effect sizes (e.g. Cohen's *d*, Pearson's *r*), indicating how they were calculated

Our web collection on statistics for biologists contains articles on many of the points above.

Software and code

Policy information about availability of computer code

Data collection	Real time PCR data was collected using StepOne software (version 2.2.2). Immunofluorescence data was collected using LAS X software (version 3.5.2.18963).
Data analysis	Immunofluorescence data was analysed using Fiji (version 2.0.0-rc-69/1.52p). Graphs and statistical analyses were done in GraphPad Prism (version 8.4.2).

For manuscripts utilizing custom algorithms or software that are central to the research but not yet described in published literature, software must be made available to editors/reviewers. We strongly encourage code deposition in a community repository (e.g. GitHub). See the Nature Research guidelines for submitting code & software for further information.

Data

Policy information about availability of data

All manuscripts must include a data availability statement. This statement should provide the following information, where applicable:

- Accession codes, unique identifiers, or web links for publicly available datasets
- A list of figures that have associated raw data
- A description of any restrictions on data availability

All relevant data are available from the authors, with the exception of individual sequencing results that could lead to loss of anonymity for patients who donated their embryos, and the codes utilized for chromosome copy number analysis, which were performed by the Foundation for Embryonic Competence (FEC), a not-for-profit entity that owns the intellectual property for genome amplification methodology and related analytical code. The source data underlying Figs 1, 2, 4, 5 and 6 and Supplementary Figs 1, 2, 3, 4, 5, 8 and 9 are provided as a Source Data file.

Field-specific reporting

Please select the one below that is the best fit for your research. If you are not sure, read the appropriate sections before making your selection.

- [x] Life sciences
- [ ] Behavioural & social sciences
- [ ] Ecological, evolutionary & environmental sciences

For a reference copy of the document with all sections, see nature.com/documents/nr-reporting-summary-flat.pdf

Life sciences study design

All studies must disclose on these points even when the disclosure is negative.

Sample size	No sample-size calculation was performed. Sample size was determined based on previous experimental evidence. For example: Bolton et al, Nature Communications, 2016 and Shahbazi et al, Nature, 2017.
Data exclusions	26 embryos were excluded from the analysis as they did not hatch.
Replication	All findings have been replicated. To ensure replication we have used embryos from multiple different patients across different independent experiments. Embryo cell numbers were manually calculated by two independent researchers. Experiments with human TSCs were independently performed by two different researchers. The exact number of independent experiments is indicated in figure legends.
Randomization	Group allocation was based on the embryo karyotype.
Blinding	Investigators were not blinded to group allocation as in several cases the karyotype could be distinguished based on embryo morphology.

Reporting for specific materials, systems and methods

We require information from authors about some types of materials, experimental systems and methods used in many studies. Here, indicate whether each material, system or method listed is relevant to your study. If you are not sure if a list item applies to your research, read the appropriate section before selecting a response.

Materials & experimental systems

n/a	Involved in the study	
	x	Antibodies
	x	Eukaryotic cell lines
x		Palaeontology
x		Animals and other organisms
	x	Human research participants
x		Clinical data

Methods

n/a	Involved in the study	
x		ChIP-seq
x		Flow cytometry
x		MRI-based neuroimaging

Antibodies

Antibodies used	Primary antibodies: mouse monoclonal anti-E-CADHERIN antibody (610182, BD Biosciences, clone 36, 1/100), goat polyclonal anti-GATA3 antibody (AF2605, R&D Systems, 1/200 dilution), goat polyclonal anti-GATA6 antibody (AF1700, R&D Systems, 1/200 dilution), rat monoclonal anti-GFP antibody (GF090R, clone GF090R, Nacalai USA, 1/1,000 dilution), mouse monoclonal anti-HLA-G (ab7759, clone MEM-G/1, 1/200 dilution), goat polyclonal anti-NANOG antibody (AF1997, R&D Systems, 1/200 dilution), mouse monoclonal anti-OCT3/4 antibody (sc-5279, Santa Cruz Biotechnology, clone C-10, 1/200 dilution), rabbit polyclonal anti-Phospho-HISTONE H3 antibody (9701, Cell Signaling Technology, 1/200), mouse monoclonal anti-PODOCALYXIN antibody (MAB1658, R&D, clone 222328, 1/500 dilution), and rabbit monoclonal anti-SYNDECAN-1 (ab128936, clone EPR6454, Abcam, dilution 1/100). Secondary antibodies: donkey anti-mouse AlexaFluor®568 (A10037, Thermo Fisher Scientific), donkey anti-rabbit AlexaFluor®647 (A31573, Thermo Fisher Scientific), and donkey anti-goat AlexaFluor®488 (A11055, Thermo Fisher Scientific).
Validation	All the antibodies used in this study have been previously used by multiple other groups in different publications.<br>- Mouse monoclonal anti-E-CADHERIN antibody (610182, BD Biosciences): Website states human, mouse, rat and dog reactivity; western blot, immunoprecipitation, immunofluorescence, and immunohistochemistry applications. Citation: Osumi D, et al. Core fucosylation of E-cadherin enhances cell-cell adhesion in human colon carcinoma WiDr cells. Cancer Science 100, 2009. DOI: 10.1111/j.1349-7006.2009.01125.x.<br>- Goat polyclonal anti-GATA3 antibody (AF2605, R&D Systems): Website states human and mouse reactivity; western blot, immunohistochemistry, and immunoflourescence applications. Citation: Phipson B, et al. Evaluation of Variability in Human Kidney Organoids. Nature Methods 16, 2019. DOI: 10.1038/s41592-018-0253-2.<br>- Goat polyclonal anti-GATA6 antibody (AF1700, R&D Systems): Website states human reactivity; western blot, immunohistochemistry, chromatin immunoprecipitation and immunoflourescence applications. Citation: Shahbazi MN, et al. Pluripotent state transitions coordinate morphogenesis in mouse and human embryos. Nature 552, 2017. DOI: 10.1038/ nature24675.

- Rat monoclonal anti-GFP antibody (GF090R, Nacalai USA): Website states western blotting, immunoprecipitation and immunoflourescence applications. Citation: Shahbazi MN, et al. Pluripotent state transitions coordinate morphogenesis in mouse and human embryos. Nature 552, 2017. DOI: 10.1038/nature24675.
- Mouse monoclonal anti-HLA-G (ab7759, clone MEM-G/1): Website states human and macaque reactivity. Citation: Guo W, et al. Decreased Human Leukocyte Antigen-G Expression by miR-133a Contributes to Impairment of Proinvasion and Proangiogenesis Functions of Decidual NK Cells. Frontiers in Immunology 2017. DOI: 10.3389/fimmu.2017.00741
- Goat polyclonal anti-NANOG antibody (AF1997, R&D Systems): Website states human reactivity; western blot, chromatin immunoprecipitation, and immunoflourescence applications. Citation: Shahbazi MN, et al. Pluripotent state transitions coordinate morphogenesis in mouse and human embryos. Nature 552, 2017. DOI: 10.1038/nature24675.
- Mouse monoclonal anti-OCT3/4 antibody (sc-5279, Santa Cruz Biotechnology): Website states human reactivity; western blot, immunohistochemistry, and immunoflourescence applications. Citation: Shahbazi MN, et al. Pluripotent state transitions coordinate morphogenesis in mouse and human embryos. Nature 552, 2017. DOI: 10.1038/nature24675.
- Rabbit polyclonal anti-Phospho-HISTONE H3 antibody (9701, Cell Signaling Technology): Website states human, mouse, rat, monkey, drosophila and yeast reactivity; western blot, immunohistochemistry, immunoflourescence and flow cytometry applications. Citation: Colicino EG, et al. Gravin regulates centrosome function through PLK1. Molecular Biology of the Cell, 2017. DOI: 10.1091/mbc.E17-08-0524.
- Mouse monoclonal anti-PODOCALYXIN antibody (MAB1658, R&D): Website states human reactivity; western blot, flow cytometry, and immunoflouresence applications. Citation: Shahbazi MN, et al. Pluripotent state transitions coordinate morphogenesis in mouse and human embryos. Nature 552, 2017. DOI: 10.1038/nature24675.
- Rabbit monoclonal anti-SYNDECAN-1 (ab128936, Abcam): Website states mouse, rat and human reactivity; western blot, immunohistochemistry, immunoflourescence, and flow cytometry applications. Citation: Wang S, et al. Syndecan-1 Suppresses Cell Growth and Migration via Blocking JAK1/STAT3 and Ras/MEK/ERK Pathways in Human Colorectal Carcinoma Cells. BMC Cancer 19, 2019. DOI: 10.1186/s12885-019-6381-y.

Eukaryotic cell lines

Policy information about cell lines

Cell line source(s)	H9 human ESCs were kindly provided by Ludovic Vallier (Stem Cell Institute, UK), under an agreement with WiCell. CT human TSCs were kindly provided by Hiroaki Okae and Takahiro Arima (Tohoku University Graduate School of Medicine, Japan)
Authentication	H9 human ESCs have been authenticated by WiCell (STR testing). CT human TSCs have been recently described, characterised and authenticated by karyotyping and gene expression analyses (Okae et al, Cell Stem Cell, 2018).
Mycoplasma contamination	Cells were routinely tested for mycoplasma contamination by PCR. All cells used in the study tested negative for mycoplasma contamination
Commonly misidentified lines (See ICLAC register)	No commonly misidentified lines were used in the study

Human research participants

Policy information about studies involving human research participants

Population characteristics	Patients that underwent In Vitro Fertilisation treatment. We did not access personal and medical information such as age or diagnosis.
Recruitment	Patients that start a cycle at IVI-RMA New Jersey are offered the possibility of donating their surplus human embryos for research. They are provided with information on the project prior to consent. Patients are not pre-selected based on any specific criteria.
Ethics oversight	The study was approved by Western IRB (Clinical IRB #20031397 and 20050731). Additional ethical approval was obtained from the Human Biology Research Ethics Committee (University of Cambridge, #HBREC.2017.24).

Note that full information on the approval of the study protocol must also be provided in the manuscript.
